# DNA Damage Repair and Bacterial Pathogens

**DOI:** 10.1371/journal.ppat.1003711

**Published:** 2013-11-07

**Authors:** Darja Žgur-Bertok

**Affiliations:** Department of Biology, Biotechnical Faculty, University of Ljubljana, Ljubljana, Slovenia; University of North Carolina at Chapel Hill School of Medicine, United States of America

## Introduction

All species require DNA repair pathways to maintain the integrity of their genomes. Bacterial damage repair mechanisms have broader roles encompassing responses to stress, long-term colonization, as well as virulence. The SOS response regulates DNA repair and damage tolerance genes in many bacterial species. This article highlights the bacterial SOS response and its significance in bacterial adaptation and pathogenesis, as well as DNA damage responses provoked by bacterial pathogens in the mammalian host.

## The SOS Response

**Figure 1:**
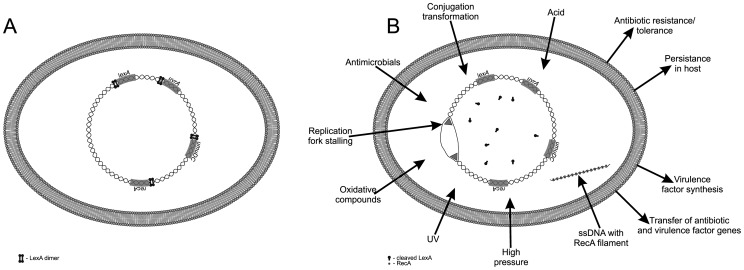
The bacterial SOS response. (A) During normal growth the LexA transcriptional repressor downregulates the SOS response genes. (B) Various endogenous and exogenous triggers induce the SOS response, resulting in drug resistance, tolerance, persistence in host, virulence-factor synthesis, and dissemination of both resistance and virulence factor genes.

The SOS response is an inducible pathway governing DNA repair that was first described in *Escherichia coli*. Two key proteins govern the SOS response: LexA (a repressor) and RecA (an inducer). In the absence of DNA damage, a LexA dimer binds to SOS boxes, a 20 base pair consensus palindromic DNA sequence, repressing transcription of a regulon encompassing more than 50 genes, including *lexA* and *recA*. Upon DNA damage, RecA is activated (RecA*) by binding to single-stranded DNA (ssDNA) to form a nucleoprotein filament. RecA* stimulates self-cleavage of LexA, leading to derepression of SOS genes. In the absence of DNA damage, basal-level expression of *lexA* ensures downregulation of the system (Figure 1) [1].

An important feature of the SOS response is its temporal control. The first genes induced are the *uvr* genes for excision of damaged nucleotides, followed by the *lexA* and *recA* genes, while genes encoding the low fidelity, error-prone repair DNA polymerases PolII (*polB*), PolIV (*dinB*), and PolV (*umuC*, *umuD*) are induced only when there is extensive, persistent DNA damage. These last-resort polymerases permit DNA replication across persistent DNA lesions that block the primary replicative DNA polymerase PolIII, but also promote an elevated mutation rate. The timing and level of expression of the individual SOS genes varies due to differences in LexA binding affinity, number and location of the SOS boxes relative to the promoter, as well as promoter strength [1].

## Triggers of the SOS Response

Various exogenous and endogenous triggers provoke the SOS response. Exogenous triggers include UV irradiation, chemicals or oxidative compounds, acids, organic mutagens, some antibiotics (e.g., fluoroquinolones such as ciprofloxacin), trimethoprim, ß lactam, and physical stressors (such as high pressure) that provoke activity of the Mrr restriction endonuclease generating DNA double-strand breaks (DBSs) [2]. Moreover, in *Vibrio cholerae* additional non-genotoxic antibiotics have been shown to induce the SOS response, namely, aminoglycosides, tetracycline, and chloramphenicol [3]. Endogenous triggers are stalled replication forks, defects following recombination or chromosome segregation, as well as metabolic by-products. Reactive oxygen species (ROS), such as superoxide radical (O_2_^−^), hydrogen peroxide (H_2_O_2_), and the highly reactive hydroxyl radical (^·^OH), are generated continuously as by-products of aerobic metabolism. ROS damage DNA, RNA, proteins, and lipids. The targeting of DNA encompasses attacks of base and sugar moieties provoking single- and double-strand breaks, adducts of base and sugar groups, and cross-links to other molecules—all lesions that block DNA replication [4].

At the molecular level, the SOS response is induced by an increase in intracellular ssDNA generated when DNA polymerase stalls at a lesion while the replicative helicase continues unwinding DNA. In addition, single-stranded DNA is generated if the replisome (an enzyme complex that replicates DNA) hops over template lesions on the leading and lagging strands [5]. Notably, ssDNA is also transiently present during two means of horizontal gene transfer: conjugation, the transfer of ssDNA via direct contact between a donor and recipient bacterial cell, and transformation, the uptake of ssDNA from exogenous double-stranded DNA. Both conjugative plasmid DNA transfer and transformation induce the SOS response [6].

## Beyond DNA Damage Repair

Whilst the SOS response was initially recognized as regulating DNA damage repair, its broader role is now well established. The SOS error-prone polymerases that enable translesion DNA synthesis also promote an elevated mutation rate that generates genetic diversity and adaptation, including the evolution of antibiotic resistance. Further, bacterial species produce a small subpopulation of transiently dormant persister cells that are tolerant of antimicrobials. Persisters play a key role in chronic bacterial infections. In *E. coli*, the SOS-controlled *tisB* gene, which encodes the toxin of the *tisB-istR1* toxin-antitoxin system, is involved in persister formation. *TisB* is a small membrane-acting peptide that decreases the proton motive force and ATP levels, which induces dormancy by shutting down cell metabolism [7].

The SOS response has also been shown to be significant in the formation of certain types of biofilms, structured communities of adherent microorganisms that exhibit increased antimicrobial resistance and increased genetic diversity [8].

Whereas both conjugation and transformation induce the SOS response, the latter in turn also activates genes involved in DNA transfer and recombination. Integrons are mobile genetic elements that have a site-specific recombination system that integrates and expresses gene cassettes with antibiotic resistance and metabolism associated functions. They frequently encode SOS-controlled integrases; therefore, the conjugation-induced SOS response triggers integron cassette recombination [9]. Further, pathogen-provoked inflammatory responses in the gut transiently increase enterobacterial colonization densities with extremely high (almost 100%) conjugation frequencies [10]. Such an increase in conjugation frequencies could transiently induce the SOS response throughout bacterial populations.

*In vivo* in a hospitalized patient, integron cassette recombination triggered by antibiotic-induced SOS response promoted the emergence of a *Pseudomonas aeruginosa* isolate highly resistant to a third-generation cephalosporin, ceftazimidine. The strain became epidemic within the hospital, spreading to other patients under antibiotic pressure [11].

Moreover, *in vitro* studies have investigated the impact of the SOS response on other mobile genetic elements and virulence-related functions. The SOS response activates integrating conjugative elements (ICEs), as exemplified by the *Vibrio cholerae* SXT that transfers and integrates into the recipient bacterial genome conferring resistance to several antibiotics [12] and by the *V. cholerae* filamentous bacteriophage CTXΦ that encodes cholera toxin [13]. The SOS response also induces pathogenicity island–encoded virulence factors in *Staphylococcus aureus* [14]. Further, SOS induction may lead to expression of prophage-encoded Shiga toxin among enterohemorrhagic *E. coli* (EHEC) [15], as well as a type III secretion system for secretion of virulence-associated factors into host cells in enteropathogenic *E. coli* [16]. In *Mycobacterium tuberculosis*, LexA regulates one of two mechanisms of DNA damage repair [17], and the error-prone α subunit of DNA-polymerase III encoded by *dnaE2* has been shown to be required for persistence during infection and for the development of antibiotic resistance.

Inhibiting induction of the SOS response could be a means of preventing the emergence and dissemination of bacterial drug resistance as well as synthesis and dissemination of some bacterial virulence factors.

## Is Competence a Stress Response that Substitutes for SOS?

Almost all bacterial phyla harbor a *lexA* gene with characteristic SOS boxes [1]. Whilst the SOS response plays an important role in the lifestyle and virulence of a number of significant pathogens, nevertheless, not all pathogens possess an SOS response. Notable pathogens that lack an SOS response are: *Campylobacter jejuni*, *Streptococcus pneumoniea*, *Streptococcus pyogenes*, *Legionella pneumophila*, *Helicobacter pylori*, *Neisseriae meningititis*, and *Neisseriae gonorherae*. In *S. pneumoniae*, *L. pneumophila*, and *H. pylori*, antibiotics provoke the induction of competence for transformation; therefore, in these species competence might substitute for SOS [18]. Competence has been hypothesized to enable DNA uptake as a nutrient to serve as a template for DNA damage repair or for genetic exchange. Nevertheless, in these three species competence is not induced by the same antibiotics, which indicates a specific fine-tuning of the response. The correlation between a lack of SOS response and competence induction by antibiotics warrants examination among other naturally competent pathogens.

## Bacteria Provoke Host DNA Damage and Repair

Whilst DNA damage repair systems play a significant role in survival and adaptation of bacterial pathogens, the latter does so by provoking chronic inflammation and/or production of genotoxins, which incite DNA damage and subsequent repair in host cells.

Upon infection, bacterial cell components stimulate host pathogen recognition receptors, provoking chronic inflammation with a constant production of ROS, reactive nitrogen intermediates, and cytokines by inflammatory cells such as macrophages and neutrophils. Chemical mediators of inflammation can damage proteins, lipids, metabolites, DNA, and RNA. Bacteria that provoke chronic inflammation have been shown to promote carcinogenesis [19]. On a global scale, chronic inflammation is presumed to be involved in 25% of all cancer cases. The best studied of these bacteria is *Helicobacter pylori*, which is associated with gastritis, peptic ulceration, gastric carcinoma, and mucosa-associated lymphoid tissue lymphoma [20]. Infection with *H. pylori* provokes DBSs, triggering a damage-signaling and repair response. Nevertheless, chronic infection with *H. pylori* also promotes downregulation of the two DNA repair mechanisms mismatch repair and base excision repair [21].

Other examples of studied associations between bacteria and chronic inflammation are the linkage of chronic carriage of *Salmonella enterica* serovar *Typhi* with a higher risk of carcinoma of the gallbladder [22] and the linkage of colonization by *Bacteriodes fragilis* with colon cancer [23].

Eukaryotic organisms possess a number of molecular mechanisms to maintain the integrity of their genomes. Among the most hazardous lesions are DSBs, since a single unrepaired DNA DSB can provoke cell death [24]. Bacterial toxins designated as genotoxins may also be a source of DSBs.

Induction of DSBs leads to activation of the DNA damage response (DDR), comprising initial sensing of DNA breaks followed by a chain of events leading to cell cycle arrest, DNA damage repair, and cell cycle resumption. Initially, DSBs activate PARP1 and PARP2, poly (ADP-ribose) polymerases that catalyze poly (ADP)-ribosylation of histones and other nuclear proteins. Subsequent recruitment of the MRN complex composed of MRE11, NBS1, and RAD50 triggers at the site of DNA damage activation of the ATM (Ataxia telangiectasia mutated) kinase via autophosphorylation. ATM activation results in phosphorylation of histone H2AX at serine 139 (γH2AX) and formation of DNA repair foci at sites of DSBs [25]. The presence of γH2AX initiates mobilization of MDC1 (mediator of DNA damage checkpoint protein 1) to recruit RNF8 and RNF168 (ring finger proteins 8 and 168) [26]. These proteins facilitate histone ubiquitination that, in turn, promotes accumulation of 53BP1 (p53-binding protein) and BRCA 1 (breast cancer gene 1) proteins [27]. ATM also activates the transcription factor p53, inducing cell cycle arrest by transcriptionally regulating the cyclin-dependent kinase inhibitor p21. Ubiquitination is thought to promote local alterations in chromatin structure facilitating DSB signaling and DNA repair. Irreversible DNA damage results in apoptosis or senescence.

So far, three types of *E. coli* genotoxins have been described: (1) the cytolethal distending toxins (CDT), also produced by other species of the *Enterobacteriaceae* including *Salmonella enterica* serovar *Typhi* [28]; (2) colibactin [29], [30]; and (3) the *E. coli* uropathogenic-specific protein [31]. Mammalian cells intoxicated with either the CDT or colibactin have been shown to activate the classical DDR [30], [32], [33].

Chronic exposure to DNA-damaging agents may cause genome instability, enhancing the risk of tumor development. *In vitro*, chronic exposure to CDT produced by *Helicobacter hepaticus* promoted induction of genome instability due to impaired activation of the DDR and cell cycle checkpoints—properties associated with tumor progression [34]. Moreover, colibactin has been shown to promote colorectal cancer. In mice, intestinal inflammation promoted alteration of microbial composition, provoking expansion of *E. coli* producing the genotoxin colibactin and subsequent tumorigenesis [35].

## Conclusions

The bacterial SOS response regulates DNA repair and restart of stalled replication forks. Induction of the SOS response affects bacterial adaptation to stress, including antimicrobial tolerance, resistance, and virulence. Blocking SOS induction could be a means of preventing the evolution of bacterial resistance and of controlling significant pathogens. In turn, bacterial pathogens in host cells provoke DNA damage and DNA repair due to chronic inflammation and/or production of genotoxins.
